# Sterilization of Metallic Instruments

**Published:** 1892-08

**Authors:** 


### Sterilization of Metallic Instruments.

After cleaning by the brush and unbleached linen, the instruments are sterilized, either by steam, hot air, or boiling water. The proceeding recommended as the most simple, is, first to brush with soap and water, then boil from ten to fifteen minutes in a one-per-cent. solution of carbonate of soda; fifty per cent. more soda should be added if the water is hard. After cooling, and during the operation, the instruments are placed in boiled water containing one-half per cent. each of carbonate of soda and carbolic acid.

After operation, the instruments are first washed in pure cold water then immersed and brushed vigorously in a one-per-cent. solution of soda to which soap has been added; then rinse and finally polish with a polishing stone and alcohol, or with a bit of chamois skin. Lastly, wash with a solution of carbonate of soda and carefully dry.

The brushes are sterilized by boiling in the soda solution for twenty or thirty minutes, and are kept immersed in a one-half-per-cent. solution of corrosive sublimate.—*Exchange.*

